# In Vivo Emergence of Pandrug-Resistant *Acinetobacter baumannii* Strain: Comprehensive Resistance Characterization and Compassionate Use of Sulbactam-Durlobactam

**DOI:** 10.1093/ofid/ofad504

**Published:** 2023-10-06

**Authors:** Mollie VanNatta, Laurie Grier, Muhammad H Khan, Paulette Pinargote Cornejo, Mohammad Alam, Samir H Moussa, Jennifer G Smith, Samuel L Aitken, Alexandre E Malek

**Affiliations:** Department of Pharmacy, Ochsner LSU Health Shreveport, Shreveport, Louisiana, USA; Division of Critical Care Medicine, LSU Health Shreveport, Shreveport, Louisiana, USA; Division of Infectious Diseases, Department of Medicine, LSU Health Shreveport, Shreveport, Louisiana, USA; Division of Infectious Diseases, Department of Medicine, LSU Health Shreveport, Shreveport, Louisiana, USA; Division of Infectious Diseases, Department of Medicine, LSU Health Shreveport, Shreveport, Louisiana, USA; Innoviva Specialty Therapeutics, Inc, an affiliate of Entasis Therapeutics Inc, Waltham, Massachusetts, USA; Department of Pharmacy, Ochsner LSU Health Shreveport, Shreveport, Louisiana, USA; Department of Pharmacy, Michigan Medicine, Ann Arbor, Michigan, USA; Division of Infectious Diseases, Department of Medicine, LSU Health Shreveport, Shreveport, Louisiana, USA

**Keywords:** acinetobacter baumannii, antimicrobial resistance, carbapenemresistant acinetobacter baumannii, multiple drug-resistant bacteria, sulbactam-durlobactam

## Abstract

The treatment of patients with infection secondary to carbapenem-resistant *Acinetobacter baumannii* with emerging cefiderocol resistance remains challenging and unclear. We present a case of in vivo emergence of pandrug-resistant *A baumannii* that was successfully treated with the compassionate use of investigational sulbactam-durlobactam–based antibiotic regimen. We also performed a longitudinal genomic analysis of the bacterial isolates and showed the development of resistance and genetic mutations over time.

*Acinetobacter baumannii* is an opportunistic pathogen that largely causes hospital-acquired infections [1–3]. *Acinetobacter baumannii* and related organisms in the *A baumannii–calcoaceticus* complex are a worldwide threat and the Centers for Disease Control and Prevention escalated carbapenem-resistant *A baumannii* (CRAB) to an urgent public health threat [4]. Due to both intrinsic factors and its ability to acquire antimicrobial resistance, *A baumannii* has become resistant to most approved antibiotics by employing several mechanisms of resistance including, but not limited to, rendering its outer membrane impermeable, modifying the antibiotic-target site, upregulating multidrug efflux pumps, and producing a wide range of β-lactamases [5]. Due to these mechanisms, there is an urgent need for effective novel antimicrobials [6]. Herein, we present a case of a patient with thermal injuries who developed septic shock due to ventilator-associated pneumonia caused by an extremely drug-resistant *A baumannii*. Initial treatment with various antimicrobials, including cefiderocol, led to the emergence of a pandrug-resistant infection. A treatment regimen that included sulbactam-durlobactam (SUL-DUR) led to resolution of the infection and subsequent discharge to outpatient care.

## CLINICAL VIGNETTE

### Case Presentation

A 44-year-old man presented to an affiliate hospital following thermal injuries with sustained full-thickness burns, covering >70% of body surface area. Patient was intubated on admission, hospital day (HD) 1, and was admitted to the intensive care unit (ICU). He had recurrent infections secondary to multidrug-resistant organisms, including *Stenotrophomonas maltophilia* (HD 11 and HD 34), *Pseudomonas aeruginosa* (HD 34), and cutaneous fusariosis (HD 23). Patient was treated appropriately for these infections with complete resolution. During ICU stay, he remained critically ill, sedated, intubated, and mechanically ventilated with eventual tracheostomy placement (HD 14). He required continuous renal replacement therapy (CRRT) and vasopressor support. Of particular concern, he developed worsening respiratory status and tenacious tracheal secretions, with chest radiographs revealing bilateral pneumonia. Cultures from bronchoalveolar lavage (BAL) were notable for CRAB on HD 63.

### Treatment and Outcome

**Figure 1.**
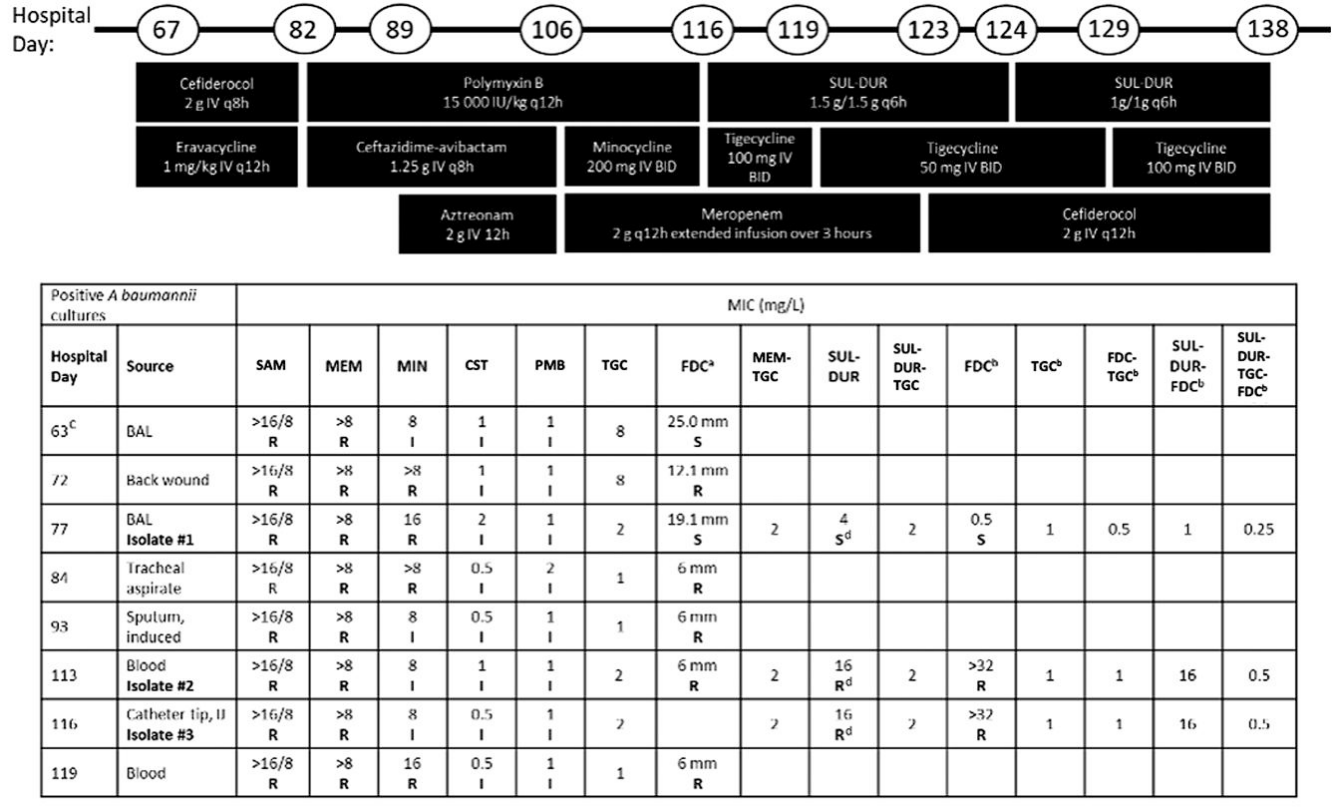
Timeline of hospital course with antibiotic usage and *Acinetobacter baumannii* isolate culture and susceptibility data. Abbreviations: BAL, bronchoalveolar lavage; BID, twice daily; CST, colistin; FDC, cefiderocol; I, intermediate; IJ, intrajugular; IV, intravenous; MEM, meropenem; MIN, minocycline; PMB, polymyxin B; R, resistant; S, sensitive; SAM, ampicillin-sulbactam; SUL-DUR, sulbactam-durlobactam; TGC, tigecycline. ^a^Sensitivity performed using disk diffusion. ^b^Sensitivity performed in iron-depleted cation-adjusted Mueller-Hinton broth. ^c^Patient was empirically treated with ceftolozane-tazobactam, vancomycin, and trimethoprim-sulfamethoxazole between hospital days 63 and 67. ^d^Agents tested in combination with SUL-DUR were titrated 1:1 with SUL and DUR was fixed at 4 mg/L, based on preliminary breakpoint of 4 mg/L.

Upon identification of CRAB, an initial treatment of cefiderocol (sensitive, zone diameter 25.0 mm) and eravacycline was initiated on HD 67 (Figure 1). While on this regimen, on HD 82 patient developed septic shock with refractory ventilator-associated pneumonia, which subsequently resulted with cefiderocol-resistant (6 mm, disk diffusion) CRAB. The susceptibility of this isolate to polymyxin B was intermediate, as there is no susceptible breakpoint for *A baumannii* (Clinical and Laboratory Standards Institute [CLSI], M100). Salvage combination therapy was initiated consisting of polymyxin B along with ceftazidime-avibactam. The tracheal aspirate culture while on this regimen grew CRAB with intermediate susceptibility to polymyxin, so aztreonam was added in case of metallo-β-lactamase–producing CRAB. Despite this regimen, the patient remained in shock and the antibiotic regimen was changed to minocycline 200 mg intravenously (IV) twice daily and meropenem 2 g extended infusion over 3 hours every 12 hours (q12h) along with polymyxin 15 000 IU/kg q12h on HD 106. He developed worsening hypotension and was found to have cardiac tamponade, status post pericardiocentesis and thoracentesis for left pleural effusion. Pericardial and pleural fluid cultures were sterile. Blood cultures yielded CRAB (Isolate 2), resistant to cefiderocol with intermediate susceptibility to polymyxin and minocycline. The treatment regimen was transitioned to cefiderocol (FDC) along with polymyxin and minocycline.

Given the continuous decline of the patient's clinical status, we submitted for emergency investigational new drug application (eIND) approval from the US Food and Drug Administration for SUL-DUR. This drug combination completed a phase 3 clinical trial (https://clinicaltrials.gov/ct2/show/NCT03894046) testing its efficacy and safety in patients with *A baumannii–calcoaceticus* complex infections and is part of an expanded access program (EAP) at Entasis Therapeutics (Waltham, Massachusetts). Once consent forms and institutional review board and eIND approval were secured, we initiated SUL-DUR therapy on HD 116. Since the patient was on active ventilator support, CRRT, and vasopressors at this time, SUL-DUR was initiated at a dose of 1.5 g/1.5 g every 6 hours (q6h) (advised CRRT dosing per the SUL-DUR EAP protocol) along with meropenem 2 g q12h extended infusion over 3 hours and tigecycline (TGC) 100 mg IV q12h. On day 3 of SUL-DUR (HD 119), liver enzymes increased slightly; therefore, the tigecycline dose was reduced to 50 mg IV q12h. The patient remained hemodynamically stable on CRRT and was off pressors. By day 4 of SUL-DUR–based combination therapy (HD 120), the patient was weaned off ventilator and was oxygenating at 99% on room air. On day 8 of SUL-DUR (HD 123), he spiked a fever of 38.0°C and repeat blood cultures 72 hours after combination therapy revealed CRAB (Table 1, blood culture from HD 119); therefore, meropenem was transitioned to cefiderocol 2 g q12h. On day 9 of treatment (HD 124), CRRT was stopped and the dose of SUL-DUR was transitioned to 1 g/1 g q6h. Susceptibility testing indicated that CRAB isolates were equivalent for tigecycline alone versus tigecycline combinations (Figure 1), so tigecycline dose was increased back to 100 mg IV q12h. Repeat blood cultures from day 16 of treatment with SUL-DUR (HD 131) remained sterile. On day 23 of treatment (HD 138), due to worsening thrombocytopenia, antibiotics were discontinued. The patient was discharged to a rehabilitation center and remained alive.

**Table 1.** Whole Genome Sequencing of the 3 Carbapenem-Resistant *Acinetobacter baumannii* Isolates

Strain ID	Isolate 1 (BAL)	Isolate 2 (Blood)	Isolate 3 (Catheter Tip)
MLST	ST^Ox^: ST-195/ST^Pa^: 2	ST^Ox^: ST-195/ST^Pa^: 2	ST^Ox^: ST-195/ST^Pa^: 2
β-lactamases present	ADC-73; TEM-1; OXA-23-like [P225T]; OXA-66	ADC-73; TEM-1; OXA-23-like [P225T]; OXA-66	ADC-73; TEM-1; OXA-23-like [P225T]; OXA-66
Other antimicrobial resistance determinants	Tet(B) (tetracycline); APH (6)-Id (aminoglycoside); APH (3″)-Ib (aminoglycoside); ArmA (aminoglycoside); ANT (3″)-IIa (aminoglycoside); Msr(E)-Mph(E) (macrolide); Sul2 (sulfonamide)	Tet(B) (tetracycline); APH (6)-Id (aminoglycoside); APH (3″)-Ib (aminoglycoside); ArmA (aminoglycoside); ANT (3″)-IIa (aminoglycoside); Msr(E)-Mph(E) (macrolide); Sul2 (sulfonamide)	Tet(B) (tetracycline); APH (6)-Id (aminoglycoside); APH (3″)-Ib (aminoglycoside); ArmA (aminoglycoside); ANT (3″)-IIa (aminoglycoside); Msr(E)-Mph(E) (macrolide); Sul2 (sulfonamide)
PBP3 allele	A515V	H370Y, A515V	H370Y, A515V
Other resistance determinants of note	…	AdeJ efflux pump [G288S]	AdeJ efflux pump [G288S]
All genomic changes compared to Isolate 1	…	PBP3 [H370Y]; AdeJ [G288S]; hypothetical protein (A1S_3656) [L69F]; hypothetical protein (A1S_1912) [T71fs]	PBP3 [H370Y]; AdeJ [G288S]; hypothetical protein (A1S_3656) [L69F]; hypothetical protein (A1S_1912) [T71fs]

Abbreviations: BAL, bronchoalveolar lavage; Oxa, oxacillinase; PBP3, penicillin-binding protein 3; ST^Ox^, ST195 by the Oxford scheme; ST^Pa^; ST2 by the Pasteur scheme.

### Susceptibility and Molecular Characterization of *A baumannii* Isolates

In addition to susceptibility testing of all positive *A baumannii* cultures, 3 isolates collected (HD 77, 113, and 116) were extensively studied, including susceptibility determination against various combinations of antibiotics and genomic characterization by whole-genome sequencing (WGS) to understand the drivers of resistance. WGS was performed using Illumina MiSeq (Supplementary material). The minimum inhibitory concentrations (MICs) for each isolate were performed using broth microdilution and determined according to the CLSI guidelines [7]. In all combinations that included SUL-DUR, the DUR concentration was maintained at 4 mg/L.

The isolate collected on HD 77 (Isolate 1) was sensitive to FDC, SUL-DUR, and combinations thereof; however, isolates collected from HD 113 (Isolate 2) and HD 116 (Isolate 3) became resistant to FDC alone (>32-fold change in MIC) as well as SUL-DUR (4-fold change in MIC, based on a preliminary susceptible breakpoint of ≤4 mg/L). Antibiotic combinations that included TGC, which does not have a susceptibility breakpoint for CRAB, remained effective in vitro. The resistance emergence between HD 77 and HD 113/HD 116 led to a pandrug-resistant infection with intermediate susceptibility to colistin. Additionally, possible exposure to suboptimal antibacterial dosing in this burn patient could have contributed to the emergence of resistance [8].

To better understand the emergence of resistance to FDC and SUL-DUR, the 3 isolates were subjected to WGS. In addition to isolate relatedness and common antimicrobial resistance determinants, antimicrobial target mutations that correlated with resistance and any other differences between the 3 strains were evaluated. Mutations and substitutions in penicillin-binding proteins (PBPs), efflux, and drug permeation genes were identified by comparison with the *A baumannii* reference strain ATCC 17978 (GenBank accession number CP000521).

Table 1 is a summary of the WGS analysis. The WGS data for these 3 isolates revealed that they are genetically related, all belonging to the same multilocus sequence types, ST195 by the Oxford scheme (ST^Ox^) and ST2 by the Pasteur scheme (ST^Pa^). All 3 strains encoded for the β-lactamase genes ADC-73 (class C), TEM-1 (class A), OXA-23-like, and OXA-66 (class D). Since all the β-lactam antibiotics used target the PBPs, especially PBP3 (cefiderocol, aztreonam, ceftazidime, meropenem, and sulbactam) analysis of the sequence of this gene was especially important. Isolate 1 had a single missense substitution in PBP3, compared to the sequence of ATCC 17978, leading to a substitution at alanine 515 (A515V). This mutation is near the active site of PBP3 and has been previously identified in both SUL-DUR and FDC susceptible and resistant isolates. In addition to this missense mutation, the subsequent isolates 2 and 3 had an additional substitution at histidine 370 (H370Y) (Supplementary Figure 1*A*). This mutation has been linked to the development of resistance to cefiderocol and carbapenems in *A baumannii* [9–11]. Its role in SUL-DUR susceptibility is currently unknown, especially in combination with the PBP3 A515V allele.

In addition to the PBP3 missense mutation, isolates 2 and 3 gained a mutation in the resistance-nodulation-division (RND) efflux pump, AdeJ (G288S) (Supplementary Figure 1*B*). This mutation, like that of the PBP3 H370Y missense mutation, has also been linked to the development of CRAB [12].

## DISCUSSION

In this report, we described a patient who had septic shock, pneumonia, and skin and soft tissue infection secondary to pandrug-resistant *A baumannii*, and was successfully treated with a SUL-DUR–based antibiotic regimen. In addition, we performed longitudinal comprehensive profiling of CRAB isolates, including WGS, to elucidate the acquired resistance mechanisms that occurred in vivo.

Current treatment options for CRAB include cefiderocol, but an optimal combination regimen has yet to be determined, with guidance recommending utilization of at least 2 active agents [13]. Sulbactam (SUL) was originally developed as a β-lactamase inhibitor; however, in *Acinetobacter* spp, SUL also has intrinsic activity, inhibiting PBP1 and PBP3 [14]. Unfortunately, due to the acquisition of β-lactamases, especially class D OXA-family β-lactamases, SUL is frequently degraded in contemporary CRAB isolates. In contrast to the commercially available β-lactamase inhibitors such as avibactam, vaborbactam, or relebactam, durlobactam is a non-β-lactam diazabicyclooctane β-lactamase inhibitor that exhibits activity against class A, C, and D β-lactamases and restores the activity of SUL in resistant *A baumannii* [15]. The combination of SUL-DUR has demonstrated both in vitro and in vivo activity against multidrug-resistant and extensively drug-resistant (XDR) *A baumannii* [15, 16]. The use of SUL-DUR in a clinical setting to date includes a phase 3 trial and 2 published cases. The ATTACK trial showed a SUL-DUR–based regimen achieved noninferiority in 28-day all-cause mortality (19% vs 32% in the colistin arm) in treating patients with CRAB compared to colistin with favorable safety profile [17]. In the first published case, a patient with pneumonia and septic shock caused by an XDR *A baumannii* was successfully treated with cefiderocol and SUL-DUR for 14 days [18]. The isolate was sensitive to cefiderocol and SUL-DUR. In another case, authors described a patient who was critically ill with XDR *A baumannii* necrotizing pneumonia and empyema who failed a 7-day course of cefiderocol and tigecycline and was successfully treated with SUL-DUR combined with meropenem for 14 days [19].

A unique feature of our report was the total duration of the SUL-DUR–based regimen, up to 23 days, in which we observed no safety issues. Additionally, we observed the generation of resistance in-therapy to FDC, as has been described previously [20, 21], with the genetic characterization of the isolates occurring in almost real time. WGS of these isolates revealed the generation of PBP3-related mutations and as our patient was exposed to cefiderocol, aztreonam, ceftazidime, and meropenem, we speculate that prior exposure to these antibiotics could have selected for the PBP3 H370Y mutation, leading to an increased MIC against SUL-DUR. Additionally, we observed a mutation in the RND efflux pump, AdeJ, whose role in resistance to FDC and SUL-DUR remains unknown. Of interest, Barnes et al demonstrated that the bacterial isolates with high SUL-DUR MICs (≥8 µg/mL) encoded either for A326V or S1010R mutations in AdeJ or H370Y or A578T mutations in PBP3 [22]. In surveillance studies published to date, resistance to SUL-DUR was due to either the presence of metallo-β-lactamases, such as NDM-1, which DUR does not inhibit, or the presence of certain PBP3 mutants for which SUL has a reduced binding affinity [23, 24]. The comprehensive susceptibility testing of various combinations, as advised by the Infectious Diseases Society of America, showed that combinations of SUL-DUR and TGC were effective in vitro and translated to this patient's recovery after nearly 138 days in the ICU [13].

An important aspect and likely successful outcome for this EAP patient was the use of the ATTACK trial protocol–driven CRRT regimen of 1.5 g SUL/1.5 g DUR q6h. Prescribers must estimate the dosage for these critically ill patients until pharmacokinetic studies can be conducted; however, it is rare that consistent recommended dose regimens become established [25]. Although we do not have drug concentrations for this patient, based on Entasis Therapeutics’ CRRT experience in the ATTACK trial, the SUL-DUR concentrations were likely sufficient to cover the 4-fold shift in SUL-DUR MIC to 16 mg/L based on exceeding systemic pharmacokinetic/pharmacodynamic exposure targets (unpublished data on file).

## CONCLUSIONS

This case describes the clinical use of SUL-DUR with a combination of antibiotics that included tigecycline and cefiderocol to successfully treat a CRAB infection with emerging cefiderocol resistance. The resistance to cefiderocol emerged during antibiotic therapy, which may have influenced resistance emergence to SUL-DUR, prior to SUL-DUR exposure. However, treatment with SUL-DUR and tigecycline led to a positive outcome for this patient and further research studies are warranted to evaluate this antibiotic combination against emergent cefiderocol-resistant *A baumannii*.

## Supplementary Data

Supplementary materials are available at *Open Forum Infectious Diseases* online. Consisting of data provided by the authors to benefit the reader, the posted materials are not copyedited and are the sole responsibility of the authors, so questions or comments should be addressed to the corresponding author.

## Supplementary Material

ofad504_Supplementary_Data

## Notes

***Acknowledgments.*** We thank Entasis Therapeutics for supplying sulbactam-durlobactam through its expanded access program. We are grateful to Dr David Altarac, Chief Medical Officer of Entasis Therapeutics, for his tremendous help and guidance throughout the treatment process including amending the protocol to use the investigational medication beyond 14 days based on the patient's clinical response.

***Author contributions.*** S. H. M. performed antimicrobial susceptibility testing and whole-genome sequencing and analysis of Isolates 1–3.

***Patient consent.*** The patient's written consent was obtained, and the design of the work has been approved by local institutional review boards.

***Data availability.*** All genome sequencing data described in this manuscript have been deposited at DNA Data Bank of Japan/European Nucleotide Archive/GenBank as part of Bioproject PRJNA988113, under the accession numbers JAUEPW000000000, JAUEPX000000000, and JAUEPY000000000. The versions described in this article are versions JAUEPW010000000, JAUEPX010000000, and JAUEPY010000000.
