# Supplementary material for: In Vivo Emergence of Pandrug-Resistant Acinetobacter baumannii Strain: Comprehensive Resistance Characterization and Compassionate Use of Sulbactam-Durlobactam
Source: Open Forum Infect Dis. 2023 Oct 6;10(10):ofad504. doi: 10.1093/ofid/ofad504 (PMC10603586; doi:10.1093/ofid/ofad504)
Supplement: ofad504_Supplementary_Data [file ofad504_supplementary_data.zip › Entasis methods_Revised_Supplement materials..docx]

***Antimicrobial susceptibility testing***

Antimicrobial susceptibility testing for isolates #1-3 were performed at Entasis Therapeutics using broth microdilution in cation-adjusted Mueller Hinton broth following CLSI methods[1], except for cefiderocol and combinations thereof where MICs were performed in iron-depleted Mueller-Hinton broth [2]. Quality control testing was performed using *Escherichia coli* strain ATCC 25922, *Pseudomonas aeruginosa* strain ATCC 27853, and *A. baumannii* strain NCTC 13304[2] . MIC values were interpreted using CLSI breakpoints for all antimicrobial agents except those for which CLSI breakpoints are not available[3] . All agents were tested as 2-fold dilutions, with agent combinations tested at a ratio of 1:1. Combinations that include durlobactam were tested with durlobactam at a fixed concentration of 4 mg/L.

***Whole-genome sequencing and analysis***

Extraction of chromosomal DNA, whole-genome sequencing, and subsequent analysis of the genomic content of each isolate was performed at Entasis Therapeutics (Waltham, MA). Chromosomal DNA was extracted from each isolate using the Promega Maxwell 16 instrument and Maxwell 16-cell DNA purification kit following the manufacturer’s protocol (Promega, Madison, WI). DNA was quantified with a Qubit 2.0 fluorometer using the double-stranded DNA (dsDNA) broad-range assay kit (Life Technologies, Grand Island, NY). DNA was diluted to 0.2 ng/μl, and a 5-μl amount was used for library generation using the Nextera XT DNA sample preparation kit and Nextera XT index primers (Illumina, San Diego, CA). The recommended procedure was followed, except that the library normalization step was omitted in favor of quantitative PCR (qPCR) library quantification. qPCR was performed on a Bio-Rad CFX96 cycler using the NEBNext Library Quant Kit (Ipswich, MA). Libraries were diluted to a standard concentration of 4 nM DNA, and 2.5 μl of each sample (8 to 12 samples, targeting 25- to 50-fold coverage) was combined and denatured with 0.1 N NaOH (final) for 5 min. The sample was diluted to 600 μl to provide a 15- to 20-pM multiplex library. Samples were sequenced on an Illumina MiSeq instrument using the V2 chemistry in a 2 × 150-bp paired-end read format.

Assembly and analysis of whole-genome sequencing was performed using CLC Genomics Workbench version 22.0 (Qiagen, Aarhus, Denmark). Fastq files were processed and analyzed as follows: duplicate sequence reads were removed, and remaining reads were trimmed for quality and minimum length (50 bp). Reads were de novo assembled at high stringency (fraction length = 0.9 and similarity fraction = 0.99) using default mismatch/insertion/deletion costs. Detection of single-nucleotide polymorphisms (SNPs) and indels was accomplished through mapping to isolate #1 using the same parameters. Quality-based SNPs were detected at a minimum frequency of 80% using default criteria.

The antimicrobial resistance genes including β-lactamase content of each strain was determined by BLAST within the CLC Genomics Workbench against an assembled database of genes curated at Entasis Therapeutics, with sequences originating from the NCBI Bacterial Antimicrobial Resistance Reference Gene Database (accession number PRJNA313047). For MLST determination, assembled contigs were exported from CLC Genomics Workbench and uploaded into the PubMLST database (https://pubmlst.org/databases/). For *Acinetobacter*, PubMLST hosts two different MLST schemes, Oxford and Institut Pasteur. The Oxford scheme (ST^ox^) assigns sequence types using the following genes: *gltA*, *gyrB*, *gdhB*, *recA,* *cpn60*, *gpi,* and *rpoD.* Alternatively, the Institut Pasteur scheme (ST^Pa^) assigns sequence types using alleles of *cpn60*, *fusA*, *gltA*, *pyrG*, *recA*, *rplB*, and *rpoB*. Sequence types from both schemes are reported.

**References:**

1. Methods for Dilution Antimicrobial Susceptibility Tests for Bacteria That Grow Aerobically. Available at: www.clsi.org.

2. Lewis II JS, Melvin Weinstein FP, Bobenchik AM, et al. M100-Ed32 February 2022 Replaces M100-Ed31 Performance Standards for Antimicrobial Susceptibility Testing Suggested Citation. 1986. Available at: www.clsi.org.P:+1.610.688.0100;F:+1.610.688.0700;E:customerservice@clsi.org;W:www.clsi.org.

3. M100 Performance Standards for Antimicrobial Susceptibility Testing A CLSI supplement for global application. Available at: www.clsi.org.
