# Supplementary figures and images for: In Vivo Emergence of Pandrug-Resistant Acinetobacter baumannii Strain: Comprehensive Resistance Characterization and Compassionate Use of Sulbactam-Durlobactam
Source: Open Forum Infect Dis. 2023 Oct 6;10(10):ofad504. doi: 10.1093/ofid/ofad504 (PMC10603586; doi:10.1093/ofid/ofad504)

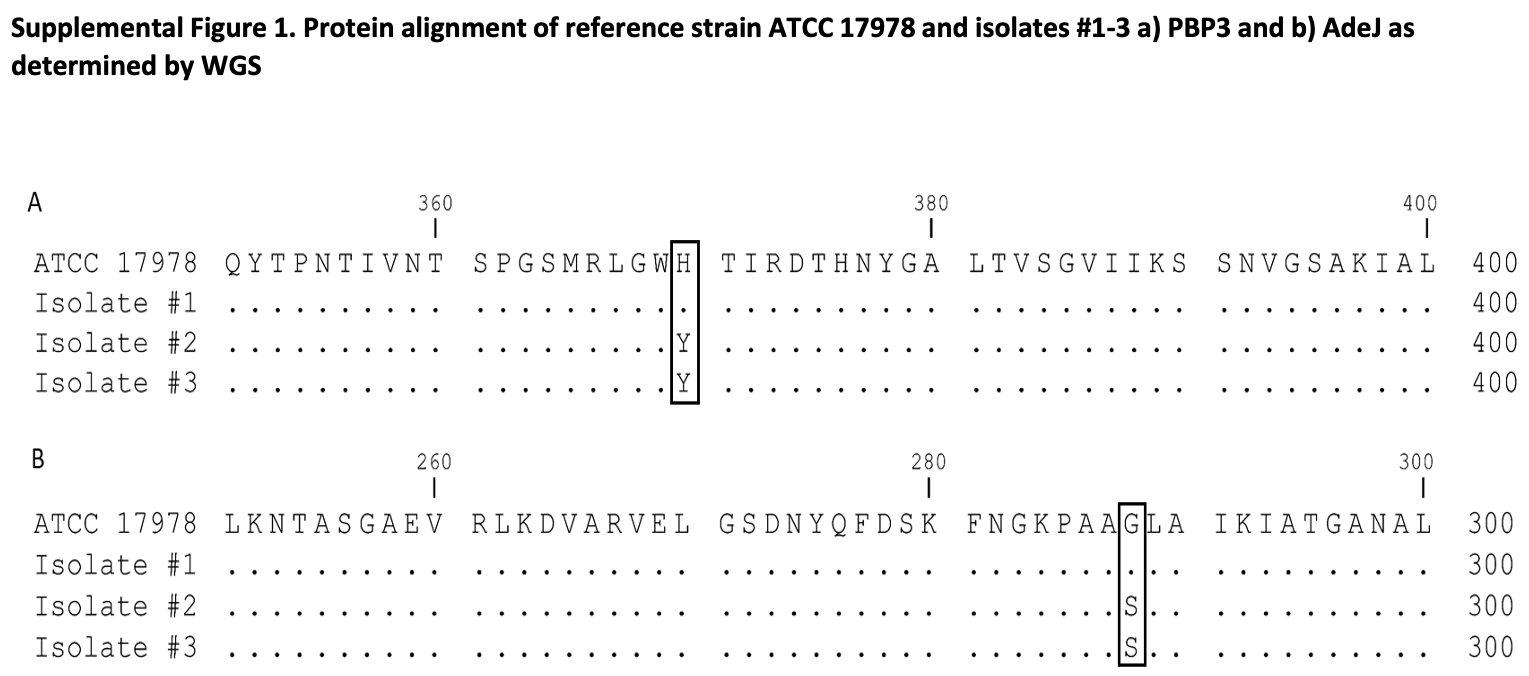

Supplement: ofad504_Supplementary_Data [file ofad504_supplementary_data.zip › Supplemental figure 1.tiff]
